# Thromboembolic Events Following Atrial Fibrillation Cardioversion and Ablation: What’s the Culprit?

**DOI:** 10.3390/medicina55080505

**Published:** 2019-08-20

**Authors:** Francesco De Sensi, Gennaro Miracapillo, Luigi Addonisio, Marco Breschi, Alberto Cresti, Pasquale Baratta, Francesco Paneni, Ugo Limbruno

**Affiliations:** 1 Cardiology Department, Misericordia Hospital, 58100 Grosseto, Italy; 2 Center for Molecular Cardiology, University Hospital, 8057 Zürich, Switzerland

**Keywords:** atrial fibrillation ablation, stroke, iatrogenic interatrial septum defect, paradoxical embolism, anticoagulant interruption

## Abstract

Stroke is a rare but possible complication after atrial fibrillation (AF) ablation. However, its etiopathogenesis is far from being completely characterized. Here we report a case of stroke, with recurrent peripheral embolism after AF ablation procedure. In our patient, an in situ femoral vein thrombosis and iatrogenic atrial septal defect were simultaneously detected. A comprehensive review of multiple pathophysiological mechanisms of stroke in this context is provided. The case underlines the importance of a global evaluation of patients undergoing AF ablation.

## 1. Case Report 

**Figure 1:**
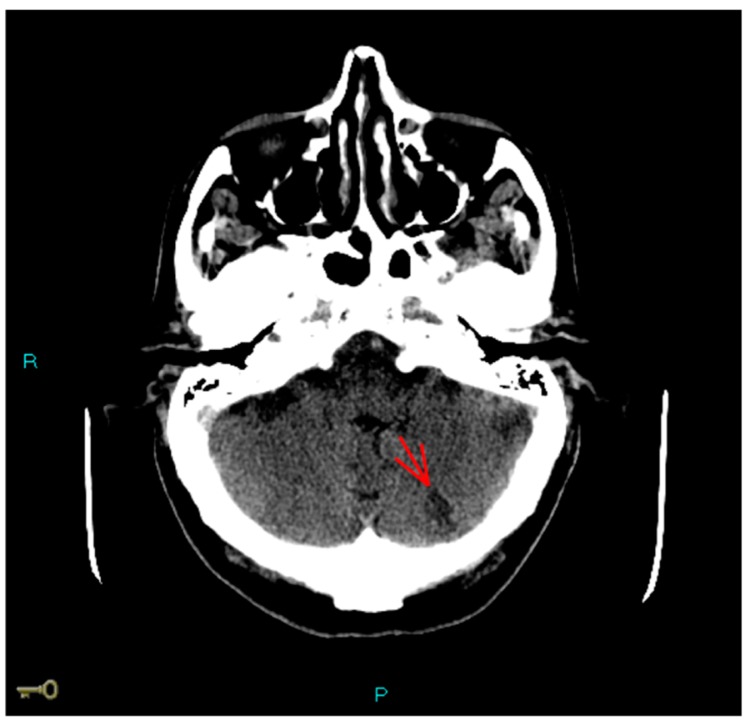
Angio-CT (computational tomography) brain scan. The exam showed an acute ischemic lesion in the left cortico-subcortical temporo-occipital area and in the left cerebellar hemisphere (last one marked with red arrow).

**Figure 2:**
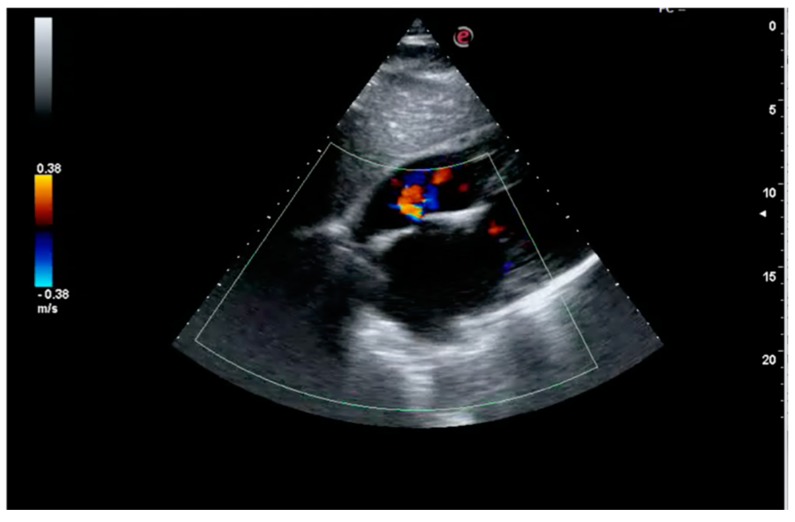
Transthoracic echocardiogram (subxiphoid view). The exam showed a clearly discernable interatrial septal defect with left-to-right shunt identified, at rest, with color doppler.

**Figure 3:**
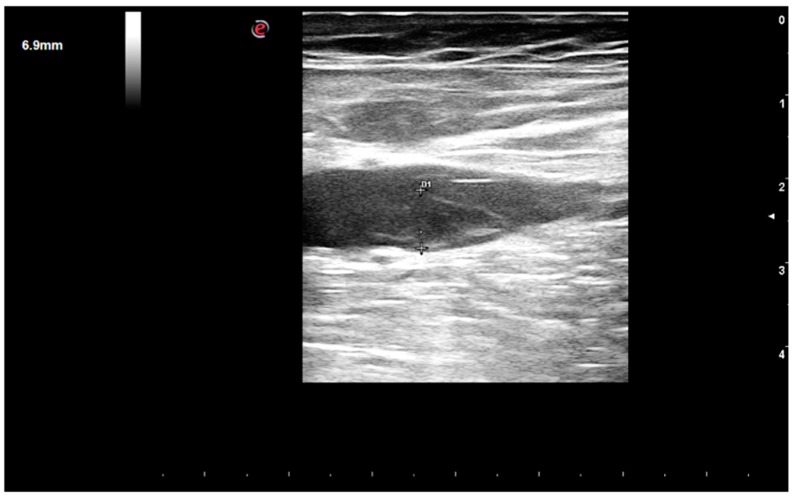
Ultrasonographic femoral scan. The exam showed in situ thrombosis of the left femoral vein which was not compressible with the probe.

A gentleman, 76 years old, was scheduled for catheter ablation of atrial fibrillation (AF) and atypical left atrial flutter in the context of symptomatic left ventricular dysfunction. He reported fatigue and exertional dyspnea, and presented persistent AF on EKG. He had a weight of 68 kg, and a height of 170 cm (BMI = 23 kg/m^2^), with high estimated thromboembolic risk (CHA_2_DS_2_VASc = 4). He was previously prescribed with anticoagulation (Dabigatran 110 mg bid), beta-blocker (bisoprolol 5 mg od), ACE-inhibitor (ramipril 5 mg od), diuretic (furosemide 50 mg) therapy. A 2D-echocardiogram documented left ventricle dilation (LVEDD (end diastolic diameter): 61 mm) with systolic dysfunction (EF (ejection fraction): 38%). A 2D-transesophageal echocardiogram (TEE) showed absence of images referable to atrial and auricular thrombosis. Single-lobe left appendage displayed reduced function with velocity peaks of 25 cm/sec. The left atrial area was 28 cm^2^. No relevant atherosclerotic plaques were found in the thoracic aorta. Written informed consent was obtained and the patient underwent radiofrequency electrical pulmonary veins isolation plus roof and mitral isthmus ablation lines during systemic intraprocedural heparinization (activation clotting time (ACT)-target: 300–350 s). Electrical cardioversion was also performed due to presence of persistent AF. The total procedural time was 180 min. Dabigatran was temporarily interrupted for 36 h across the procedure and the patient was discharged the next day. After one week he was admitted to the emergency department for sudden dyspnea, being hospitalized for acute heart failure. At admission the EKG showed sinus tachycardia, while chest X-ray depicted bilateral alveolar edema. During hospitalization, after achieving hemodynamic stabilization, the patient suffered aphasia and space-time disorientation with near loss of consciousness. The Angio-CT (computational tomography) showed hypodense lesions in the left cortico-subcortical temporo-occipital area and in the left cerebellar hemisphere as showed in Figure 1. Carotid and vertebral arteries were free from hemodynamic atherosclerotic plaques. Symptoms completely disappeared after two days and at the 24 h CT scan control, the lesions were stable, in the absence of hemorrhagic transformation. After a few days, the patient complained left limb pain and an acute distal embolism was diagnosed. A new transthoracic echocardiogram revealed a further deterioration of left ventricular ejection fraction (EF: 30%) with no evidence of intraventricular thrombosis and a clearly discernable interatrial septal defect with left-to-right shunt, this was likely attributable to the trans-septal puncture performed during the ablation (Figure 2). Ultrasonography of the groin region documented in situ not compressible left femoral vein thrombosis (Figure 3). Non fractioned heparin infusion was administered with complete resolution of both the arterial embolic occlusion and venous thrombosis. After a few days, oral anticoagulation with apixaban was initiated and the patient was discharged. At the six months follow-up, he presented with mild cognitive impairment, which persisted overtime till the last visit.

## 2. Case Discussion 

The case illustrates an uncommon complication after atrial fibrillation (AF) ablation manifested with recurrent embolic events: A stroke and a leg embolism. Although stroke is a well-known described complication after AF ablation, the etiopathogenetic mechanisms underlying this complication are yet to be completely characterized. Here, we summarize and discuss all the potential factors involved in this undesirable complication. 

### 2.1. Radiofrequency Lesion Set and Ablation “Per Se” 

Evidence from non-randomized studies has shown that AF catheter ablation may reduce stroke risk, when successful. Among 361,913 patients with AF of the Swedish Patient Registry, catheter ablation was associated with a lower risk of stroke (HR = 0.69) and mortality (HR = 0.50). These results were even more significant in patients with CHA_2_DS_2_-VASc score ≥2 (HR = 0.39) [1]. Especially in patients with CHA2DS2-VASc score of ≥2 (83% of 3953 patients) Saliba and colleagues found a reduction in stroke rate in the ablation group compared to the non-ablated group (HR = 0.61) [2]. On this ground, Hunter et al. demonstrated, in an international multicenter registry of 1273 patients, that freedom from AF was associated with stroke-free survival (HR = 0.30) [3]. However, when discussing the possibility of a catheter ablation procedure for AF treatment, physicians should clearly make their patients aware about a periprocedural stroke risk which is approximately 0.5–1% [4]. Thromboembolic risk is directly related to the amount of radiofrequency lesions applied in the left atrial cavity. In fact, radiofrequency produces colliquative necrosis, thus leading to endothelial dysfunction and activation of the Virchow triad. Hence, during ablation, tissue involvement is directly related to an increased embolic risk [5]. An approach adding linear or complex lesion sets to pulmonary vein isolation (PVI) did not demonstrate an increase in freedom from AF recurrences, thus the standard endpoint during the first procedure should be PVI alone [6]. In our case, extensive left atrial ablation was performed with PVI plus tracing of two ablation lines along the roof and the mitral isthmus. Such ablation strategy was due to the presence of atypical left atrial flutter as well as of persistent atrial fibrillation. Although stroke is considered an uncommon complication after AF ablation, a growing body of evidence is consistently reporting asymptomatic or subclinical ischemic lesions in up to 41% of patients [7,8]. An elegant Italian study by Gaita and colleagues analyzed postprocedural brain magnetic resonance imaging (MRI) of 232 consecutive patients with paroxysmal or persistent atrial fibrillation who underwent radiofrequency left atrial catheter ablation. Techniques used were PVI or PVI plus linear lesions plus atrial defragmentation. A clinical cerebrovascular accident occurred in only 1 patient. However, brain MRI returned positive for new embolic lesions in 33 patients. Cardioversion (CV) during the procedure was associated with an increased risk of 2.75 (95 confidence interval, 1.29–5.89; *p* = 0.009) [9]. Our patient underwent electrical CV during the ablation due to the presence of persistent AF at the beginning of the procedure. It has been recognized that CV is related to thromboembolic events “per se”, independently by the ablation procedure. In patients undergoing TEE-guided cardioversion, patients on direct oral anticoagulants (DOACs), such as dabigatran and apixaban, experienced low incidence of thromboembolic events during follow-ups (0.6% and 1.1%, respectively), similar to warfarin, with a favorable trend of bleeding safety profile [10,11]. The highest risk period after CV is the following week, which would be a suitable timeline for our patient considering the stroke and the peripheral embolism. 

Finally, it seems that techniques other than radiofrequency, such as cryoballoon based one-shot ablation and duty-cycled phased radiofrequency ablation (PVAC) are not free from silent cerebral embolisms, suggesting other mechanisms (like air embolism) could play a pivotal role in the physiopathology of these subclinical findings [12,13,14]. 

### 2.2. Management of Anticoagulant Therapy in the Periprocedural Period

Ablation was performed in January 2017. The patient had been prescribed Dabigatran 6 months before. We decided to perform TEE due to the patient’s high thromboembolic risk (CHA_2_DS_2_VASc = 4). Indeed, despite optimal oral anticoagulation with DOACs, left atrial (LA) thrombus was detected in the left appendage (LAA) in >3.6% of AF patients undergoing catheter ablation in the real world. In this setting higher CHA_2_DS_2_VASc (*p* = 0.02), but not the type of DOAC, significantly predicted the presence of LA thrombus [15]. 

Dabigatran has been available in Italy since 2014. At the time of ablation there were no clear guidelines on appropriate periprocedural ablation management of such a new kind of drug. On the contrary, evidence available on uninterrupted warfarin showed reduction in bleeding and thromboembolic complications [16]. Since we used all the available tools in order to reduce bleeding complications (i.e., ultrasound guided femoral veins puncture, intracardiac echocardiography, contact force sensing catheters) [17,18], we felt confident to minimize dabigatran interruption. In fact, the last assumption was in the morning of the day before, and first retake was in the evening of the day of the procedure (36 h, total interruption time). Despite this short interruption and the use of heparinization during the procedure (target activation clotting time ACT = 300–350 s), we should consider this anticoagulation break as a putative factor implicated in the patient’s recurrent embolic events. Indeed, the 2017 expert consensus statement on AF Ablation provide a Class I recommendation for performing the procedure with uninterrupted dabigatran (Class I, LOE A) or rivaroxaban (Class I, LOE B-R), and a 2A recommendation for the other Xa inhibitors for which specific clinical studies had not been performed at the time [5]. These recommendations derived from the results of the RE-CIRCUIT trial which was a head-to-head comparison between uninterrupted dabigatran and uninterrupted warfarin in patients undergoing AF ablation. The incidence of major bleeding was significantly lower with dabigatran than with warfarin (5 patients (1.6%) vs 22 patients (6.9%)). No strokes/TIA (transient ischemic attack) occurred in the dabigatran arm, while there was one TIA in the warfarin group. Idarucizumab, the specific reversal agent, was never used during the study [19]. Two years before, Cappato and colleagues published the results of the VENTURE-AF trial, comparing uninterrupted rivaroxaban vs uninterrupted warfarin. Complications (a major bleeding event, one ischemic stroke, and one vascular death) occurred only in the warfarin group [20]. More recently consistent evidences were provided also for apixaban and edoxaban. The AEIOU trial, published in 2018, randomized 300 patients undergoing AF ablation to uninterrupted versus minimally interrupted (holding 1 dose) periprocedural apixaban. A retrospective cohort of patients treated with uninterrupted warfarin at the same centers was matched to the apixaban-treated subjects for comparison. There were no stroke or SE events observed in all groups. The rates of clinically significant, major bleeding were similar for all apixaban patients compared with the matched warfarin group [21]. Finally, in 2019 Hohnloser et al. published results from the ELIMINATE-AF trial, which confirmed the safety and efficacy of uninterrupted edoxaban vs vitamin K antagonists (VKAs) in the same setting. Among 553 patients undergoing AF ablation, brain magnetic resonance imaging was performed in 177 subjects to assess silent cerebral infarcts. There was one ischaemic and one haemorrhagic stroke, both in patients on edoxaban. Cerebral microemboli were detected in 13.8% (16) of patients who received edoxaban and 9.6% (5) of patients in the VKA group (*p* = ns) [22]. Based on these clinical trials, it is now clear that a strategy of performing AF ablation on patients receiving uninterrupted anticoagulation can be performed safely and will minimize the risk of thromboembolic events. Finally, international guidelines state in the absence of controlled trial data, anticoagulation management after AF ablation should follow general recommendations (i.e., on the basis of CHA_2_DS_2_-VASc score), regardless of the presumed rhythm outcome [23].

### 2.3. Iatrogenic Interatrial Septal Defect, In Situ Thrombosis and Paradoxical Embolism 

The diagnosis in our patient, of simultaneous iatrogenic interatrial septal defect (IASD) and in-situ thrombosis, is rather unique. These are two well characterized phenomena that have rarely been discovered together in this setting. Real incidence of IASD after AF ablation is under debate. Older studies, using transesophageal echocardiography (TEE), reported up to 19% rate during follow-up [24,25,26,27]. 

More recently, other rates have been described (5.6% following a first procedure and 2.2% following a second procedure) [28]. 

The risk of persistent IASD is in part related to the tools, technologies, and approaches used for catheter ablation. For example, the incidence of IASD at 1-year follow-up following cryoballoon ablation procedure for PVI is significantly higher in front of radiofrequency procedures [29,30,31]. After a single-puncture, using the robotic navigation system, an IASD was detected in 38 of 40 (95%) patients one day after the ablation. At 6-months follow up, the IASDs were closed only in 30 of 39 (78.9%) patients. The authors also addressed that persistent IASDs are not associated with an increased rate of paradoxical embolism or with relevant shunting [32].

On the other side, the real incidence of in situ asymptomatic femoral thrombosis after AF ablation is unknown. Asymptomatic deep venous thrombosis (DVT) formation, following sheath placement for electrophysiological studies (EPS) in general, were detected in up to 16–44% of patients. In contrast, symptomatic DVTs are much lower (0.5–0.8%) [33]. In 2004, Chen and colleagues reported a significant incidence (17.6%) of non-occlusive DVT after multiple sheath placements for EPS. Nonetheless, in the study, all venous thrombi were non-occlusive and asymptomatic. None of the femoral veins developed occlusive DVT [34]. Although there are weak supporting data, it is reasonable to conclude that limiting the number and the size of femoral vein sheaths on the same side can minimize thrombosis risk. Despite the fact that there are no large prospective or randomized trials, prophylactic heparin administration during the procedure may be considered on an individual basis for right chamber ablations, particularly for longer procedures, or in high-risk patients [35]. Large emboli migrated from leg veins can lodge in the right ventricle [36], whereas smaller emboli are likely to pass unimpeded to the pulmonary arteries. The occurrence of pulmonary embolism following EP procedures has previously been reported, especially in patients with a thrombophilic state [37]. Moreover, two cases of floating atrial thrombi following EP studies were successfully treated with thrombolysis in asymptomatic patients [38].

To the best of our knowledge, there are no reported cases of paradoxical embolism following AF ablation where in situ thrombosis and iatrogenic atrial septal defect are detected simultaneously. Indeed, DVT developed despite fully systemic heparinization during the procedure and minimal oral anticoagulation interruption.

## 3. Conclusions

In conclusion, we report a case of stroke and peripheral embolism after atrial fibrillation ablation procedure. In our patient an in situ femoral vein thrombosis and iatrogenic atrial septal defect were simultaneously detected. We highlighted and discussed each etiopathogenetic mechanism underlying this clinical condition. The case encourages a critical clinical and instrumental evaluation in the management of such undesirable complications.
